# Supplementary material for: Causal Relationship between Aging and Anorexia Nervosa: A White-Matter-Microstructure-Mediated Mendelian Randomization Analysis
Source: Biomedicines. 2024 Aug 16;12(8):1874. doi: 10.3390/biomedicines12081874 (PMC11351342; doi:10.3390/biomedicines12081874)
Supplement: Supplementary file 1 [file biomedicines-12-01874-s001.zip › Supplementary Figure/Figure S4/Singe SNP plot of MR analysis for TL to ubm-b-2012.pdf]

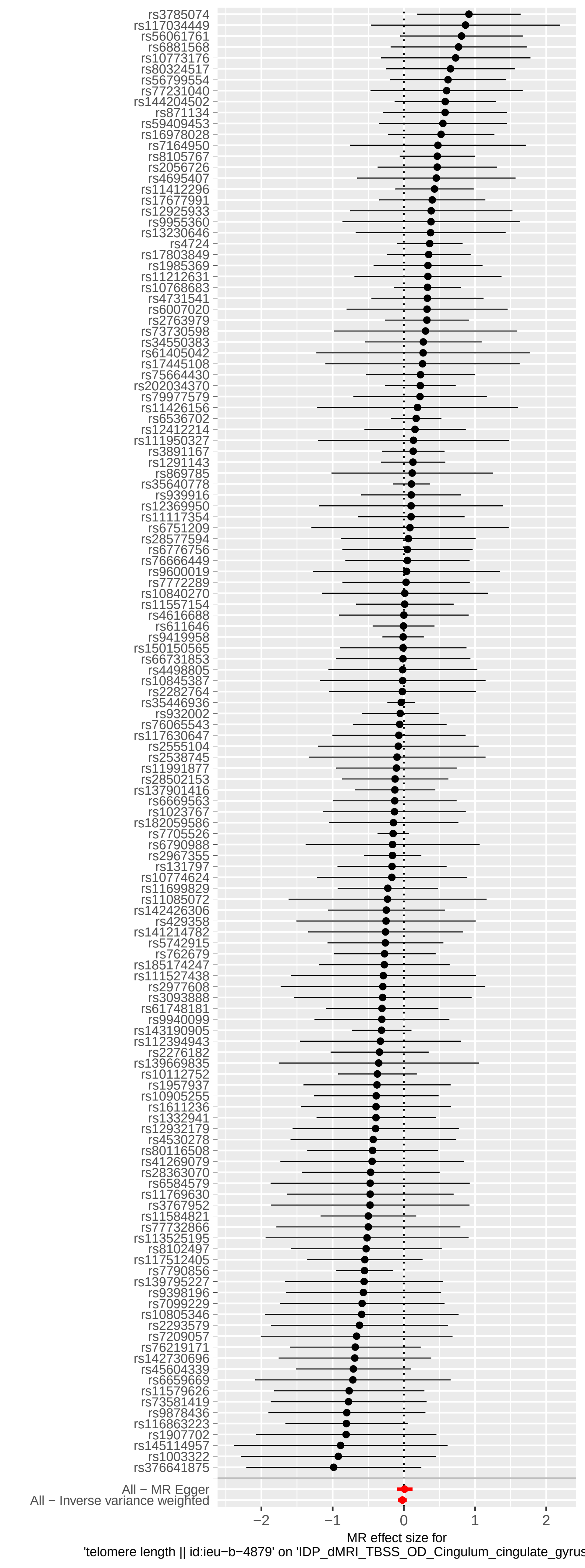
rs3785074
rs117034449
rs56061761
rs6881568
rs10773176
rs80324517
rs56799554
rs77231040
rs144204502
rs871134
rs59409453
rs16978028
rs7164950
rs8105767
rs2056726
rs4695407
rs11412296
rs17677991
rs12925933
rs9955360
rs13230646
rs4724
rs17803849
rs1985369
rs11212631
rs10768683
rs4731541
rs6007020
rs2763979
rs73730598
rs34550383
rs61405042
rs17445108
rs75664430
rs202034370
rs79977579
rs11426156
rs6536702
rs12412214
rs111950327
rs3891167
rs1291143
rs869785
rs35640778
rs939916
rs12369950
rs11117354
rs6751209
rs28577594
rs6776756
rs76666449
rs9600019
rs7772289
rs10840270
rs11557154
rs4616688
rs611646
rs9419958
rs150150565
rs66731853
rs4498805
rs10845387
rs2282764
rs35446936
rs932002
rs76065543
rs117630647
rs2555104
rs2538745
rs11991877
rs28502153
rs137901416
rs6669563
rs1023767
rs182059586
rs7705526
rs6790988
rs2967355
rs131797
rs10774624
rs11699829
rs11085072
rs142426306
rs429358
rs141214782
rs5742915
rs762679
rs185174247
rs111527438
rs2977608
rs3093888
rs61748181
rs9940099
rs143190905
rs112394943
rs2276182
rs139669835
rs10112752
rs1957937
rs10905255
rs1611236
rs1332941
rs12932179
rs4530278
rs80116508
rs41269079
rs28363070
rs6584579
rs11769630
rs3767952
rs11584821
rs77732866
rs113525195
rs8102497
rs117512405
rs7790856
rs139795227
rs9398196
rs7099229
rs10805346
rs2293579
rs7209057
rs76219171
rs142730696
rs45604339
rs6659669
rs11579626
rs73581419
rs9878436
rs116863223
rs1907702
rs145114957
rs1003322
rs376641875
All − MR Egger
All − Inverse variance weighted
−2
−1
0
1
2
MR effect size for
'telomere length || id:ieu−b−4879' on 'IDP_dMRI_TBSS_OD_Cingulum_cingulate_gyrus
